# Internal Nasal Valve Incompetence Is Effectively Treated Using Batten Graft Functional Rhinoplasty

**DOI:** 10.1155/2013/734795

**Published:** 2013-04-04

**Authors:** J. C. Bewick, M. A. Buchanan, A. C. Frosh

**Affiliations:** Department of ENT, East and North Hertfordshire NHS Trust, Lister Hospital, Corey's Mill Lane, Stevenage SG1 4AB, UK

## Abstract

*Introduction*. Internal nasal valve incompetence (INVI) has been treated with various surgical methods. Large, single surgeon case series are lacking, meaning that the evidence supporting a particular technique has been deficient. We present a case series using alar batten grafts to reconstruct the internal nasal valve, all performed by the senior author. *Methods*. Over a 7-year period, 107 patients with nasal obstruction caused by INVI underwent alar batten grafting. Preoperative assessment included the use of nasal strips to evaluate symptom improvement. Visual analogue scale (VAS) assessment of nasal blockage (NB) and quality of life (QOL) both pre- and postoperatively were performed and analysed with the Wilcoxon signed rank test. *Results*. Sixty-seven patients responded to both pre- and postoperative questionnaires. Ninety-one percent reported an improvement in NB and 88% an improvement in QOL. The greatest improvement was seen at 6 months (median VAS 15 mm and 88 mm resp., with a *P* value of <0.05 for both). Nasal strips were used preoperatively and are a useful tool in predicting patient operative success in both NB and QOL (odds ratio 2.15 and 2.58, resp.). *Conclusions*. Alar batten graft insertion as a single technique is a valid technique in treating INVI and produces good outcomes.

## 1. Introduction

Internal nasal valve incompetence (INVI) is an often overlooked cause of nasal obstruction which in turn can mistakenly be attributed to other anatomical variations such as septal deviation and turbinate hypertrophy. It is characterised by the collapse during inspiration of the upper lateral cartilages; a narrowing of the angle between the dorsal septum and upper laterals can also contribute but to a lesser extent. Functional rhinoplasty procedures for INVI have evolved over the past decade. A wide range of techniques exists in which surgeons use alar batten and/or spreader grafts, butterfly grafts, lateral crural strut grafts, alar rim grafts [1], and lateral suspension sutures. Alar batten grafts were first shown by Toriumi et al. [2] as an effective technique for correction of internal nasal valve collapse. Since then they have been widely used, but a reliable case series has yet to be published.

Case-based series assessing specific techniques are not widely available. When single-technique series are published, they often concentrate on the cosmetic outcomes. Series are usually small [3] and involve several operating surgeons [4] without specific outcomes. Recent papers have called for a single technique by a single operating surgeon to be assessed [5]. 

Predictors of which patients will benefit from alar batten graft functional rhinoplasty are also not well described. Without actively examining for INVI, a clinician may not identify the cause of symptoms which many patients find to have a considerable impact on their quality of life. Various scoring systems such as the NOSE scale do not specifically relate to the symptoms of INVI [6]. While clinicians could carry out rhinomanometry to assess the severity of INVI, how this relates to symptom improvement following an invasive procedure is not clear. As functional rhinoplasty procedures continue to grow, there is a need for a simple yet effective method for assessing patients preoperatively. We present a large case series of one specific technique, alar batten grafts, to treat INVI; this includes the use of nasal strips as a positive predictor in the success of surgery. All procedures were performed by the senior author with long-term followup.

## 2. Methods

One hundred and seven consecutive patients with INVI seen in the routine Ear, Nose, and Throat clinics of the East and North Hertfordshire NHS Trust district were included in this prospective study over a seven-year period. All patients were assessed pre-operatively by the senior author for clinical confirmation of INVI. This assessment included verification of nasal airflow restriction, worse on inspiration, in either newly presenting patients or those not previously improved by other surgical procedure(s). Patients were assessed for a positive Cottle's manoeuvre for evidence of INVI on inspiration. They were also assessed for airflow improvement after decongestion with xylometazoline 0.1% spray whereby those cases with nasal congestion secondary to inferior turbinate hypertrophy who improved with decongestion were excluded. Patients were asked to complete a pre-operative questionnaire detailing previous procedures, nasal trauma, and the use of nasal strips during pre-operative assessment.

All patients were asked to try adhesive nasal strips prior to surgery and asked if the strips had improved nasal breathing. This was not compulsory and therefore not all patients took part in this intervention.

Visual analogue scales (VAS) (0–100 mm) were used to assess the degree of nasal blockage (where 0 is the least possible blockage and 100 the worst possible) and quality of life (where 0 is “severe impact on quality of life” and 100 “excellent quality of life”). The scales were marked with the extreme of symptoms but not a numbered scale. This was completed at pre-operative assessment and then postoperatively at the appropriate follow-up appointments, at 1 week, 6 weeks, 6 months, and 12 months. Patients were seen at varying intervals depending on clinical need. 

Pre- and post-operative VAS scores were evaluated. The Wilcoxon signed-rank test was used to assess the nonparametric data at the post-operative time intervals indicated above. Some patients were followed up for longer periods of time with the VAS but numbers were too low to be clinically relevant. 

### 2.1. Operative Technique

It was the senior author's practice to use batten grafts only as the single method for correction of INVI. The aim of this technique was to strengthen the lateral walls, thus preventing internal nasal valve collapse, rather than increasing the cross-sectional space or reconstructing the external nasal valve. All patients were consented for functional rhinoplasty with graft harvesting from septal or auricular cartilage.

### 2.2. Preparation

Following preparation of the nose with Moffat's solution, a Killian's incision was made. Subsequent raising of the submucoperichondrial flaps allowed access to the quadrilateral cartilage. If septal deviation was present, a septoplasty was performed to improve nasal patency. Cartilage was harvested via a standard submucous resection technique. The cartilage was assessed for size and quality intraoperatively and kept in 0.9% saline solution until required. If the amount of septal cartilage harvested was deemed insufficient in amount and/or quality, auricular cartilage was harvested instead. Conchal cartilage was obtained following marking the antihelical fold through the posterior aspect of the pinna with Bonney's blue ink applied with a 16 G gauge (green) needle. An incision was made on the posterior surface of the pinna along the line of the blue marks, conchal cartilage harvested, and the incision closed with dissolvable sutures.

### 2.3. Alar Batten Graft Insertion

**Figure 1:**
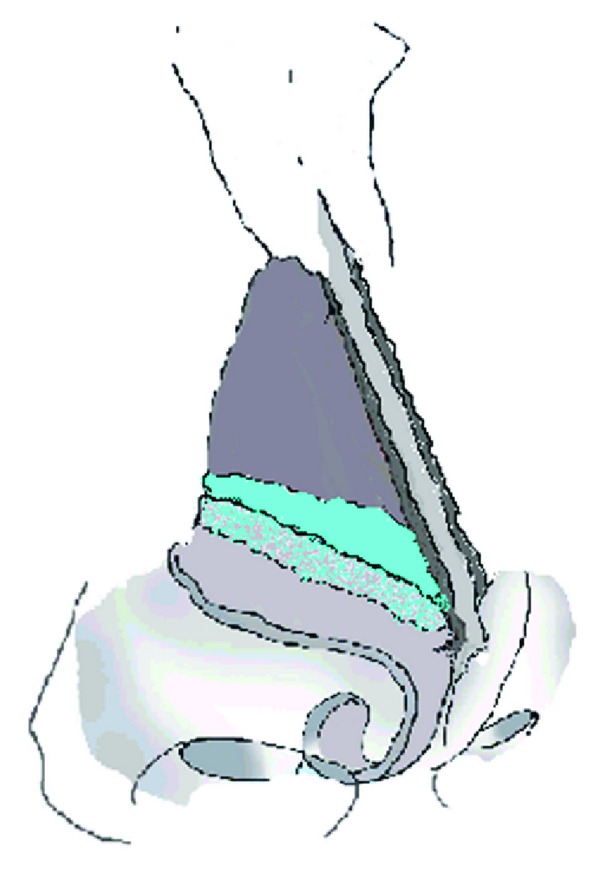
Alar batten graft insertion. The graft can be seen superficial and cephalic to the upper lateral cartilage thereby supporting the upper lateral cartilage and preventing collapse.

Grafts were fashioned in strips from the harvested cartilage, measuring 12–20 mm by 6–8 mm. This was dependent on the dimension of the lateral nasal wall as evaluated by the senior author and also partially related to the quality of quadrilateral cartilage available. To raise pockets for graft insertion, an incision was made at the dorsal edge of the septum at the junction of the upper lateral cartilages (ULCs). The caudal edges of the ULCs were dissected just to the edge of the piriform aperture and a pocket fashioned. Following this, the cartilage grafts were adjusted to fit the dissected pockets within the “scroll” area between the ULCs and lateral crura of the lower lateral cartilages (see Figure 1). The scroll area itself is left intact with the graft laying cephalic to the s-shaped scroll. Once inserted, the grafts were secured with 5/0 Ethilon to the septum to prevent migration. The Killian's incision was closed with dissolvable sutures. 

## 3. Results

One hundred and seven patients underwent functional rhinoplasty to treat INVI. Sixty-two patients were females. 

Ninety-four patients (87.9%) responded to a pre-operative questionnaire. Eighty-five patients (79.4%) responded to post-operative questionnaires reevaluation of their symptoms using the exact VAS assessment tool used pre-operatively. Post-operative followup (and repeat of VAS assessment) was performed according to patient clinical need, and hence patients were assessed at different post-operative time points. For those patients who completed both pre- and post-operative questionnaires (67 patients), 91% of patients reported long-term improvement in nasal blockage on VAS using the Wilcoxon signed-rank test; *P* values for this test can be seen in Table 1.

### 3.1. Improvement over Time

**Figure 2:**
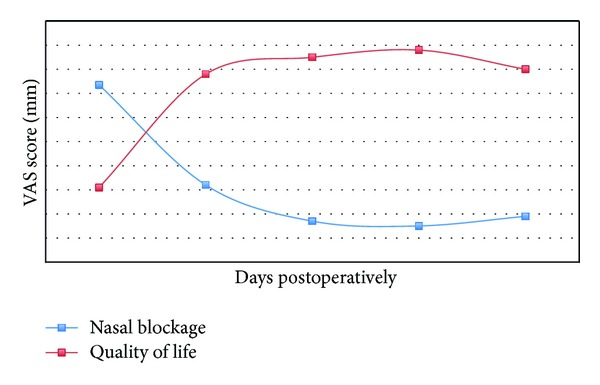
Median nasal breathing scores over time in months (0 = no patency, 100 = full patency) and quality of life scores over time (0 = severe impact on quality of life, 100 = no impact on quality of life).

Nasal breathing: prior to surgery, the median VAS of nasal blockage (0 mm = no blockage, 100 mm = full blockage) was 73.5 (range 18–100). Post-operatively, this improved to 32 mm, 17 mm, 15 mm, 19 mm, and 25 mm for 1-week, 6-week, 6-month, 12-month and 18-month periods respectively (see Table 1 for ranges). This is displayed in Figure 2, and 3 patients felt there was a decline in nasal patency following surgery; 2 patients experienced an initial improvement then decline. One patient experienced no change in symptoms. 

Quality of life scores: similar to nasal patency scores, quality of life scores also improved post-operatively (albeit based on a slightly lower figure of 59 patients, 88%). The largest improvement was seen within the first 6 months following surgery. Two patients felt there was no improvement in QOL following surgery, 3 patients felt there was a decline, and 3 patients felt an initial improvement followed by decline.

### 3.2. Postoperative Complications

Of the 107 patients initially included in the study, one patient developed unsightly nostril asymmetry as a result of inadvertent hitching of the graft to the lower lateral cartilage necessitating a subsequent cosmetic surgical treatment. One patient developed a post-operative infection characterised by persistent pain and redness. This settled with oral antibiotics. In 5 cases, the graft was seen to have resorbed significantly necessitating further surgery using auricular cartilage graft replacement for the original septal batten grafts; revision grafting was successful in all occasions. One patient required removal of the graft at a second procedure as the initial procedure had failed to improve nasal airflow and the graft was deemed unsightly.

### 3.3. Do Nasal Strips Predict Future Surgical Success?

Following initial examination, patients were asked to try nasal strips prior to surgery. Fifty-four patients did so and of them 46 patients found that the nasal strips improved their symptoms. Following surgery, 49 patients within this group had improvement in post-operative “nasal blockage” scores (odds ratio 2.152 (95% confidence interval 0.303–4.001)). This was similar to “quality of life scores” with odds ratio of 2.580 (95% confidence interval 0.566–4.594).

## 4. Discussion

Alar batten grafting as a single procedure is a useful technique for treating INVI. The technique concentrates on improving the structural integrity of the internal nasal valve rather than increasing its cross-sectional area. This is thought to be the key in treating this condition [7] and probably accounts for the improvements in nasal obstruction score experienced by patients. Symptomatic INVI is due to significant collapse of the ULCs during nasal inspiration either during exercise or at rest. Alar batten grafts improve the rigidity of the ULCs preventing collapse during negative upper airway pressure during inspiration (with septoplasty where necessary, although all patients have undergone a degree of submucous resection for graft harvesting or during previous septal surgery at a separate date). As collapse of the internal nasal valve is a dynamic process, the alar batten graft is not required to improve the overall nasal patency. 

Our study has provided evidence that the procedure is helpful to patients not only for short-term 6 weeks but also for longer periods of time. It is difficult within a busy NHS department (from which the majority of patients were recruited) to follow up NHS patients a long term due to a variety of factors the most important of which are demanding on-clinic follow-up slots, frequent change in clinical staff and patient migration to other areas. We readily admit that longer followup would be desirable but we feel that the current data as presented is at least useful. Another area for criticism is the use of a VAS to assess quality of life; while not recognised as a specific health utility to assess this aspect of surgical outcome, it does provide a useful insight into patient satisfaction in regard to their nasal surgery with minimal time input for the user. We felt that just assessing nasal blockage as the sole outcome would be too biased opinion in the technique and we wanted a more holistic view of outcomes but appreciated that patients did not always have time to complete lengthy questionnaires. 

INVI has been treated with a range of different techniques. Several different types of grafts have been used such as lateral crural strut grafts and spreader grafts [1], sometimes in combination with alar batten grafts [8]. It is difficult to assess the effectiveness of alar batten graft placement alone. This paper allows evaluation of this single technique. The assessment of a single technique for purely functional reasons is not widely available. Graft insertion is often combined with other procedures such as inferior turbinate reduction [9] and can concentrate on cosmetic outcome rather than functional results [10] or the correction of external nasal valve correction. Further INVI surgery is often performed by several surgeons within an institution [11]. This case series adds valuable data to previously published work [5] and is one of the only case series performed by one operating surgeon. This provides consistency in technique unlike other series published. 

Many patients presenting with INVI have had previous nasal surgery with unsuccessful results/outcomes. We feel that this is often due to mis-diagnosis leading to unsatisfactory outcomes following routine nasal procedures. We would advise clinicians to include Cottle's manoeuvre when performing routine examination of the nose for nasal obstruction although it should only be used in quiet inspiration as applicable to routine respiration. We would advise this during general inspection of the nose and prior to decongestion with xylometazoline spray. In our experience, patients with INVI often find immediate relief during this manoeuvre although it can be nonspecific and should not be used as the sole basis for recommending surgery for INVI.

Nasal strips are a useful positive predictor of those who will benefit from alar batten grafts. By using nasal strips in pre-operative assessment, the clinician can identify those patients who will improve both in terms of nasal obstruction and in quality of life (odds ratio 2.15 and 2.58, resp.). Ideally all patients in our study would have used the strips but as these are not provided on the NHS and come therefore at the patients' own cost, we did not insist on patients' participation. Gruber et al. have documented the use of nasal strips to evaluate “inspiratory nasal function” [12] but to our knowledge the relationship between this and post-operative outcomes has not previously been documented. With such a simple, noninvasive test, clinicians now have an excellent diagnostic tool in the investigation of such patients.

## 5. Conclusions

- Clinicians should use Cottle's manoeuvre to examine all patients with nasal obstruction prior to application of nasal decongestants but must recognise that its results are user dependent and that oversupporting the upper lateral cartilages will give a false impression of possible surgical outcomes.
- Alar batten graft to provide structural integrity of the internal nasal valve is a reliable technique with good outcomes in both nasal patency and quality of life scores. This technique has been refined by the senior author and shows that alar batten grafts alone (rather than in combination with spreader grafts) are adequate.
- Improvement in nasal patency with nasal strips is a good predictor of those patients who will benefit from alar batten grafting.

## Figures and Tables

**Table 1:** Nasal blockage and quality of life VAS scores with Wilcoxon signed-rank test *P* values. Eleven patients completed the questionnaire at both 6 and 12 months.

	Median nasal blockage VAS score in mm (range)	Nasal blockage *P*-value	Median overall quality of life VAS score in mm (range)	Quality of life *P* value (Wilcoxon signed-rank test)
Preoperatively (*n* = 85)	73.5 (18–100)		31 (3–87)	
1 week (*n* = 18)	32 (5–90)	<0.005	78 (4–96)	<0.005
6 weeks (*n* = 53)	17 (1–94)	<0.005	85 (3–100)	<0.005
6 months (*n* = 29)	15 (0–90)	<0.005	88 (16–100)	<0.005
12 months (*n* = 27)	19 (2–100)	<0.005	80 (20–100)	<0.005

## References

[1] Kim DW, Rodriguez-Bruno K (2009). Functional rhinoplasty. *Facial Plastic Surgery Clinics of North America*.

[2] Toriumi DM, Josen J, Weinberger M, Tardy ME (1997). Use of alar batten grafts for correction of nasal valve collapse. *Archives of Otolaryngology*.

[3] Millman B (2002). Alar batten grafting for management of the collapsed nasal valve. *Laryngoscope*.

[4] Rhee JS, Poetker DM, Smith TL, Bustillo A, Burzynski M, Davis RE (2005). Nasal valve surgery improves disease-specific quality of life. *Laryngoscope*.

[5] Spielmann PM, White PS, Hussain SSM (2009). Surgical techniques for the treatment of nasal valve collapse: a systematic review. *Laryngoscope*.

[6] Stewart MG, Witsell DL, Smith TL, Weaver EM, Yueh B, Hannley MT (2004). Development and validation of the Nasal Obstruction Symptom Evaluation (NOSE) scale. *Otolaryngology*.

[7] Weber SM, Baker SR (2010). Alar cartilage grafts. *Clinics in Plastic Surgery*.

[8] Khosh MM, Jen A, Honrado C, Pearlman SJ (2004). Nasal valve reconstruction: experience in 53 consecutive patients. *Archives of Facial Plastic Surgery*.

[9] Most SP (2006). Analysis of outcomes after functional rhinoplasty using a disease-specific quality-of-life instrument. *Archives of Facial Plastic Surgery*.

[10] Byrd DR, Otley CC, Nguyen TH (2000). Alar batten cartilage grafting in nasal reconstruction: functional and cosmetic results. *Journal of the American Academy of Dermatology*.

[11] Cervelli V, Spallone D, Bottini JD (2009). Alar batten cartilage graft: treatment of internal and external nasal valve collapse. *Aesthetic Plastic Surgery*.

[12] Gruber RP, Lin AY, Richards T (2011). Nasal strips for evaluating and classifying valvular nasal obstruction. *Aesthetic Plastic Surgery*.

